# Quantification of IGF-1 Receptor May Be Useful in Diagnosing Polycythemia Vera–Suggestion to Be Added to Be One of the Minor Criterion

**DOI:** 10.1371/journal.pone.0165299

**Published:** 2016-11-03

**Authors:** Jen C. Wang, Guanfang Shi, Stacey Baptiste, Maryna Yarotska, Hemant Sindhu, Ching Wong, Madhumati Kalavar, Vladimir Gotlieb, Andrei Bandarchuk, Hui Chen

**Affiliations:** Division of Hematology/Oncology, Brookdale University Hospital Medical Center, Brooklyn, NY, United States of America; Queen's University Belfast, UNITED KINGDOM

## Abstract

Endogenous erythroid colony (EEC) formation is one of the minor criteria for diagnosing polycythemia vera (PV) according to 2008 WHO diagnostic criteria. But EEC requires bone marrow aspiration and sophisticated laboratory procedures; therefore, practically it is rarely used to diagnose PV. Insulin-like growth factor 1 receptor (IGF-1R) was found to be constitutively phosphorylated and was responsible for the EEC formation in PV; therefore, we measured IGF-1R levels in the peripheral blood of 26 PV patients and compared them with those of 33 patients with secondary polycythemia and 29 normal controls. Among the PV patients, 16 were treated with only phlebotomy, 9 received hydroxyurea, and 1 was treated with ruxolinitinib. We found that PV patients treated with only phlebotomy had significantly higher IGF-1R levels than did those PV patients treated with hydroxyurea or ruxolinitinib. None of the secondary PV patients or normal controls had elevated IGR-1R levels, while 14 of 16 (87%) PV patients had significantly elevated IGF-1R levels. The new 2016 WHO has eliminated EEC as a minor criterion for diagnosing PV, but there are still some cases that cannot be definitively diagnosed by the current criteria. Therefore, we suggest that quantifying the IGF-1R level in peripheral blood by flow cytometry to replace EEC as the minor criterion for diagnosing PV.

## Introduction

The WHO criteria 2008 for diagnosing PV use the JAK2 mutation as one of the major criteria for diagnosing PV [1], which can help establish a diagnosis in most cases of PV [2,3]. The minor criteria included endogenous erythroid colony (EEC) formation *in vitro*, bone marrow (BM) trilineage proliferation, and serum erythropoietin (EPO) levels. EEC was first reported in the culture of BM from PV patients without added EPO [4], which was later found in the culture of peripheral blood (PB) as well [5,6]. This test has been the specific test for diagnosing PV since 1980 prior to the JAK2 era [5–8]. However, this test has been found to be impractical and is seldom used to diagnose PV. The reasons have been methodological, since the greatest specificity and sensitivity have been observed when BM, as opposed to PB, was used [9]; moreover, the *in vitro* clonal assay is neither standardized nor widely available [10]. Therefore, EEC has been deleted in the proposed criteria [11] and adopted by the new 2016 WHO criteria [12] for diagnosing PV.

Increased tyrosine phosphorylation of the insulin-like growth factor 1 receptor (IGF-1R) in circulating mononuclear cells (MNC) of PV patients was reported by Mirza et al. [13]. It was also found that IGF-1, but not EPO, is responsible for EEC formation in PV [14,15]. Hence, we revisited the IGF-1R pathway in PV and found increased IGF-1R expression by flow cytometry in nearly 90% of patients with PV but not in secondary polycythemia. Because the newly revised criteria still cannot cover all the cases of PV, we suggest replacing EEC formation with PB IGF-1R level measured by flow cytometry as one of the minor criteria for diagnosing PV.

## Materials and Methods

### Patients

All myeloproliferative neoplasm (MPN) patients were diagnosed according to 2008 WHO criteria. PB was obtained from patients with written informed consent; the protocol was approved by the IRB of Brookdale University Hospital. Twenty-six patients with PV (16 received only phlebotomy (untreated); 9 were treated with hydroxyurea and 1 with ruxolitinib), 33 with secondary polycythemia (23 were secondary to heavy smoking, 5 were secondary to testosterone injection, and 5 with high EPO levels and BM morphology negative for trilineage hyperproliferation), and 29 normal volunteer controls were studied. The studies were done from January 2013 until December 2015. The clinical features of the 16 untreated PV patients are listed and their clinical features are presented in Table 1. Most patients had JAK2 V617F mutation. One patient was diagnosed based on increased red blood cell mass, BM trilineage hypercellularity, and low EPO level. S1 Table, we showed the clinical data on the patient with PV who were treated with hydroxyurea or ruxolitinib.

**Table 1:** Characteristics of Phlebotomized Only PV patients.

Patient	Age/gender	JAK -2	WBC/Platelet (x10^9^/ml)	Spleen (CM)	HX of Thrombosis	IGF-1R
1	73/M	28.7%*	12.3/756	11	N	38.08
2	78/M	positive	10.0/430	11	N	323.8
3	70/F	positive	9.0/425	11	N	35.2
4	72/M	positive	11.2/450	11	N	461.7
5	71/F	positive	9.0/433	11	N	324.9
6	72/M	67%	18.2/288	18	N	983
7	51/F	positive	20.5/732	11	N	350
8	61/M	positive	19.5/691	11	N	414
9	74/M	positive	13.6/873	11	Y	505.9
10	69/F	86%	12.1/489	16	N	459.4
11	65/F	Negative**	5.5/170	11	N	202
12	69/F	91%	29.5/896	16	N	353.5
13	70/F	37%	4.8/354	11	N	1082
14	69/F	8%	18.7/677	11	N	374.03
15	89/F	positive	26/226	11	N	182
16	81/M	20%	13.0/153	11	N	369.5

* denotes percentage of allele-burden of JAK2V617F

** Diagnosis was made by the bone marrow and increased of RBC mass.

### Flow Cytometry

MNC were isolated from PB by gradient centrifugation with Ficoll-Paque. MNC were then washed with buffer and incubated with fluorophore-conjugated primary antibody or isotype controls for 30 min at room temperature and analyzed by FACS Calibur System (BD Biosciences, San Jose, CA). PE-conjugated antibody against human IGF-1R was purchased from R&D Systems (Minneapolis,MN). APC-conjugated antibody against human CD33 and FITC-conjugated antibodies against human CD14 and CD34 were purchased from BD Pharmingen. Median fluorescence intensity was calculated using FlowJo software.

## Statistical Analyses

Data were summarized by median and interquartile range. The Kruskal-Wallis test was used to test the difference of distribution among patient groups. Pairwise comparisons between each group post Kruskal-Wallis test were performed with Dunn's test. To define a cutoff value of IGF-1R to predict patient's diagnosis as PV or secondary polycythemia, logistic regression was used to model the data, and the cutoff was chosen based on the value that provided the best classification.

## Results

**Fig 1:**
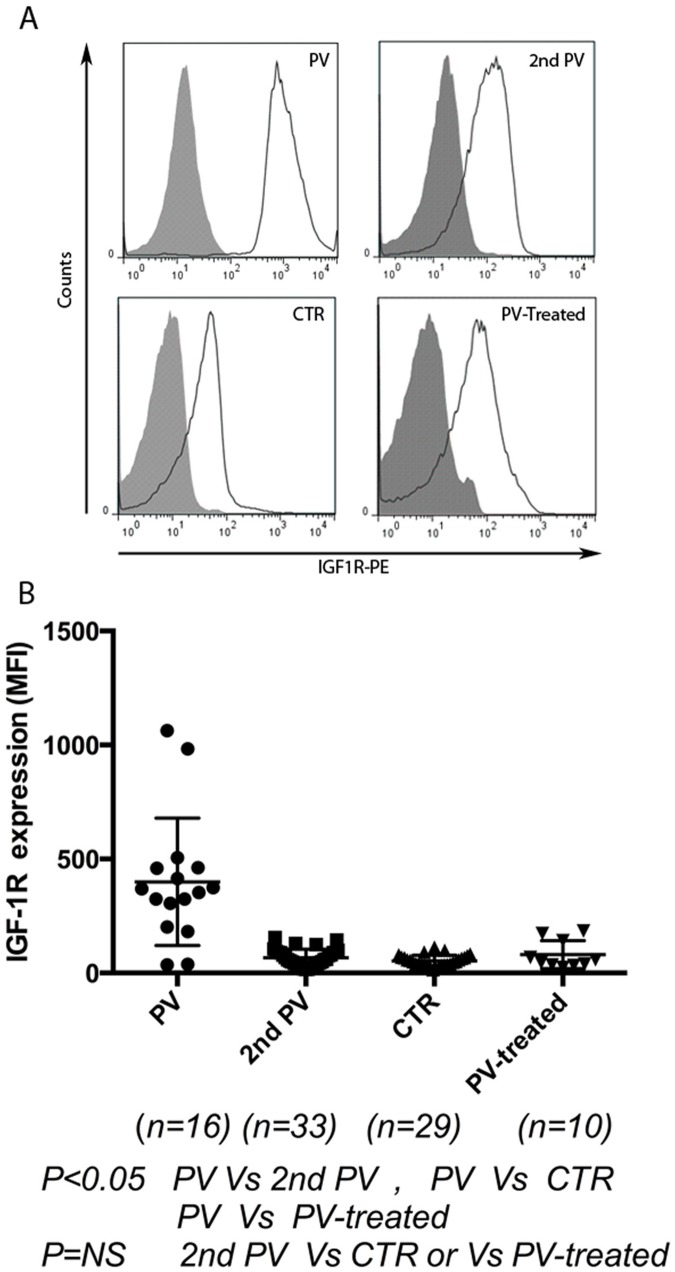
IGF-1R expression is significantly increased in patients with polycythemia vera. A) Representative flow cytometry analysis of IGF-1R (measured by MFI) in patients with untreated PV (received only phlebotomy), secondary polycythemia, normal controls, and treated PV (treated with hydroxyurea or ruxolitinib). B) Untreated PV patients have significantly increased IGF-1R (measured by MFI), results were expressed as median, interquartile range in PV (361.5, 227.8–461.1), secondary polycythemia (58.13,15.46–90.43), controls (49.20,14.63–113.5), and treated PV (52.29,23.89–149.3) (*P*<0.05).

IGF-1R was measured by median fluorescence intensity and expressed as median and interquartile range. As shown in Fig 1, untreated PV patients had significantly higher IGF-1R (361.5, 227.8–461.1) than did secondary polycythemia patients (58.13, 15.46–90.43), normal controls (49.20, 14.63–113.5), and treated PV patients (52.29, 23.89–149.3) (*P*<0.05). Logistic regression showed that IGF-1R was a statistically significant (*P* = 0.003) predictor of a patient's group (PV or secondary polycythemia). A cutoff value of 163 was determined from the logistic regression to predict a patient's group; an IGF-1R≥163 suggested that a patient belonged to the PV group. In our cohort, 14 of 16 PV patients were diagnosed based on IGF-1R≥163 and; while 2 of 16 patients with JAK2V617F-positive PV had lower values, the sensitivity of this test was 87.5% and specificity was 100%. Significantly elevated IGF-1R values were found in PV patients, while no secondary polycythemia patients had high IGF-1R. Therefore, we demonstrated that diagnosing PV can be achieved by assaying IGF-1R levels with flow cytometry of PB without doing BM biopsy. The procedure is much easier and quicker than EEC tests, which need sophisticated laboratory procedures and take longer to perform.

**Fig 2:**
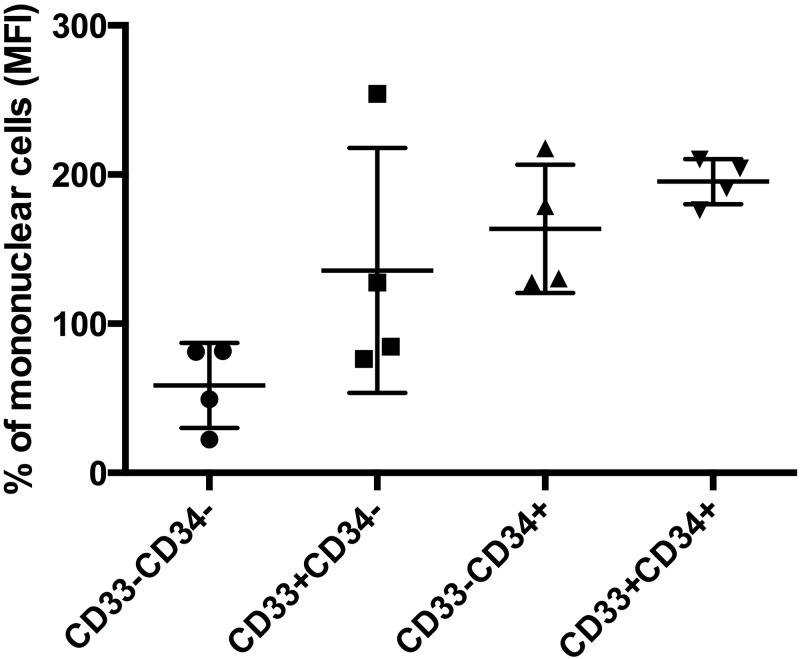
IGF-1R expression is mostly from CD34^+^ and CD33^+^cell population. Four PV patients with elevated IGF-1R were studied. Flow cytometry analysis was done to investigate which cell populations produce IGF-1R. Setting IGF-1R (MFI) of mononuclear cell population values as 100%, CD33^-^CD34^-^ cells were found to be 58±14%; CD33^+^CD34^-^, 135.7±41%; CD33^-^CD34^+^, 163.6±21.5%; CD33^+^CD34^+^,195.3±7.5%. Therefore, IGF-1R expression was mostly from CD34^+^ and CD33^+^ cell populations.

To elucidate which cell population contributed to the elevation of IGF-1R MFI, four PV patients with elevated IGF-1R were studied. As shown in Fig 2, with IGF-1R values of MNC population set at 100%, IGF-1R of CD33^-^CD34^-^ cells were 58.59±14.23%; CD33^+^CD34^-^ cells were 82.07±41.03; CD33^-^CD34^+^ cells were 163.6±21.5%; and CD33^+^CD34^+^ cells were 195.3±7.5%. Therefore, increased IGF-1R expressions in PV were mostly from the CD33^+^CD34^+^ cell population, not from the CD33^-^CD34^-^ cell population.

**Fig 3:**
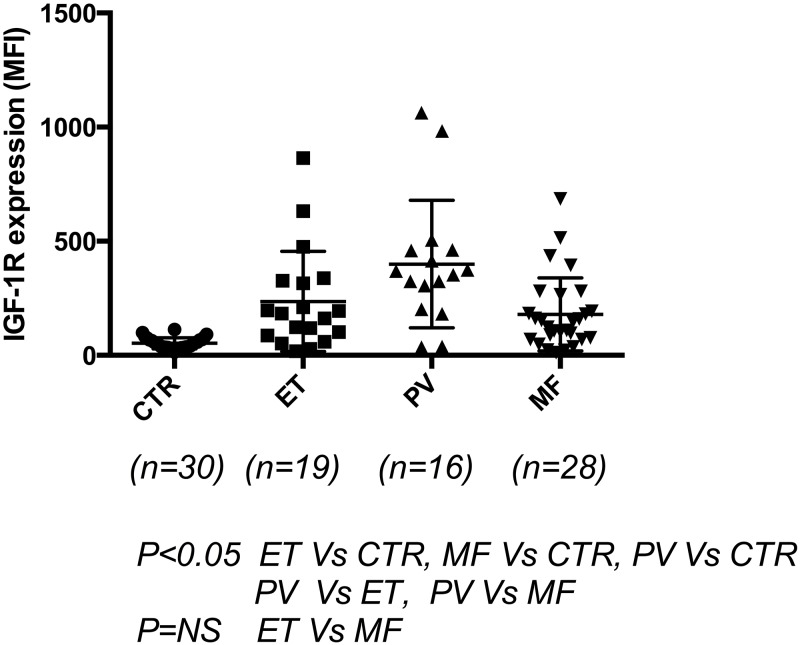
IGF-1R expression in other Ph(-) MPN patients. IGF-1R expression was measured by flow cytometry in 19 patients with essential thrombocythemia (ET), 28 myelofibrosis (MF) patients including 5 post-ET-MF, 5 post-PV-MF, and 18 primary myelofibrosis (PMF). The patients who were treated with hydroxyurea and ruxolitinib were excluded. The results showed that ET and MF patients had significantly elevated IGF-1R levels compared with controls; ET (183.0, 86.8–327.0) (n = 19) and MF (135.1, 73.01–247.6) (n = 28) patients had significantly elevated IGF-1R levels compared with controls (47.75, 34.50–68.79) (n = 30) (*P*<0.05). PV patients had elevated levels compared with ET and MF patients, while no significant difference was found between ET and MF patients.

To elucidate further the use of IGF-1R by flow cytometry to diagnose other MPN diseases, 19 patients with essential thrombocythemia (ET), 28 myelofibrosis (MF) patients including 5 post-ET-MF, 5 post-PV-MF, and 18 primary myelofibrosis (PMF) patients were compared with PV patients and controls. The patients who were treated with hydroxyurea and ruxolitinib were excluded. The results are presented in Fig 3: ET patients (183.0, 86.8–327.0) (n = 19) and MF patients (135.1, 73.01–247.6) (n = 28) had significantly elevated IGF-1R levels compared with controls (47.75, 34.50–68.79) (n = 30), and PV patients appeared to have more significantly elevated levels than did ET or MF patients. To correlate JAK2 positive vs. negative in relation to IGF-1R levels in all MPN patients; although JAK2^+^ patients had more elevated values (160.6, 47.15–325.4) (n = 46) than did JAK2^-^ patients (115.2, 66.61–280.6) (n = 25), no statistically significant correlation was found (results not shown).

## Discussion

Barbui and Tefferi [11] proposed revising WHO diagnostic criteria for PV and adopted by WHO in 2016 [12] as follows: 1) increased RCM, and/or Hb>16.5 g in men, >16 g in women or Hct>49% in men, >48% in women; 2) BM morphology consistent with WHO criteria; and 3) presence of JAK2 mutation; minor criterion: serum EPO levels. This proposal eliminates EEC and emphasizes BM findings and RCM.

BM biopsy was emphasized as the major criterion in the new WHO 2016 criteria for PV. it is characterized as showing hypercellularity for age with trilineage growth (panmyelosis) including prominent erythroid, granulocytic, and megakaryocytic proliferation with pleomorphic, mature megakaryocytes (differences in size). This characteristic bone marrow finding for diagnosing PV has been published in many reports [16–22]. But in some cases, BM examination still needs expert hematopathologists to read the results correctly, and the results may not be reproducible due to interobserver variation. A critical attitude concerning the value of BM examinations for discriminating PV from other subtypes of MPDs, as well as from reactive erythrocytosis or secondary polycythemia, has been expressed [23–26]. In the Polycythemia Vera Study Group (PVSG), an analysis of BM morphology in 281 PV patients followed for more than 9 years [23] showed that 13% of the patients did not have increased marrow cellularity or megakaryocyte hyperplasia at diagnosis. Thiele et al. also reported that of 334 patients with erythrocytosis, 4% patients could not be clearly differentiated into primary vs. secondary PV [24]. Thus, for 4–13% of patients, BM biopsy will not suitably differentiate PV from secondary erythrocytosis. For EPO levels, subnormal values were recorded in around 60% of PV patients and, in turn, normal values were found in 20% of unquestioned cases with BM morphology typical of PV [27]. Therefore, we feel that, in some small percentage of cases, PV still cannot be definitively diagnosed by BM or serum EPO levels in the cases in which JAK2, CALR, or MPL gene mutations were negative.

In our small cohort of patients, we could clearly diagnose PV in 87% of cases, and none of the secondary erythrocytosis patients had elevated IGF-1R by flow cytometry; one patient with negative JAK2V617F mutation also had trilineage proliferation of bone marrow, increased RCM, and low serum EPO who was diagnosed as PV also had elevated IGF-1R values as other PV patients. We believe that, in a small percentage of cases, assaying IGF-1R by flow cytometry can be added to the definitive diagnosis of PV. Thus, we suggest that measuring IGF-1R in the PB samples is a simple, easy, not an invasive test, (like BM biopsy) and to be added to the minor criteria for diagnosing PV.

We also found other Philadelphia chromosome -negative MPN (*Ph-MPN)* patients (hydroxyurea- and ruxolitinib-naïve ET and MF patients) had significantly increased IGF-1R relative to controls (Fig 3). Our ongoing studies will further analyze whether this can also help to differentiate the diagnosis between primary ET and secondary ET and MF cases.

Cross-talk between IGF-1R and the JAK2-V617F mutation has been demonstrated [28]. Therefore, IGF-1R levels were compared between JAK2 V617F mutation positive and negative MPN patients; no statistical significance was found. Two ET patients with a CALR-positive mutation and one with an MPL mutation were also found to have highly elevated IGF-1R expression, and two other CALR-positive ET patients were found to have normal IGF-1R values as controls. Hence, it appears that elevated IGF-1R is not correlated to JAK2V617F mutation status.

A low percentage allele burden of JAK2^+^ (<37%) was found in some patients in our series (Table 1). Different methods in measuring allele burden could accounted for the difference [29]; real-time PCR methods are less sensitive than direct sequencing, assessing alle expression (AS-PCR), or pyrosequencing. Some of the samples were performed by a commercial lab in 2010 where real-time PCR methods were used which could have explained the relative lower values than other series.

## Supporting Information

S1 Table Characteristics of hydroxyurea /ruxolitinib treated PV patients. (DOCX)
