# Supplementary material for: Quantification of IGF-1 Receptor May Be Useful in Diagnosing Polycythemia Vera–Suggestion to Be Added to Be One of the Minor Criterion
Source: PLoS One. 2016 Nov 3;11(11):e0165299. doi: 10.1371/journal.pone.0165299 (PMC5094699; doi:10.1371/journal.pone.0165299)
Supplement: S1 Table — (DOCX) [file pone.0165299.s001.docx]

| **Table S1. Characteristics of hydroxyurea /ruxolitinib treated PV patients** | | | | | | |
| --- | --- | --- | --- | --- | --- | --- |
| Patient* | Age/gender | JAK -2 | WBC/Platelet (x109/ml) | Spleen (CM) | HX of Thrombosis | IGF-1R |
| 1 | 72/F | positive | 3.6/350 | 11 | N | 38.8 |
| 2 | 70/M | positive | 4.0/210 | 11 | N | 67.9 |
| 3 | 58/M | positive | 6.0/200 | 11 | N | 36.14 |
| 4 | 68/M | positive | 15.8/401 | 11 | N | 58.5 |
| 5 | 84/F | 33.6%** | 13.6/896 | 11 | N | 32.6 |
| 6 | 86/F | 91% | 16.6/589 | 16 | N | 173 |
| 7 | 54/M | positive | 17.2/835 | 11 | N | 46.07 |
| 8 | 64/F | positive | 5.0/386 | 11 | N | 23.89 |
| 9 | 67/F | 44.70% | 16.8/351 | 11 | N | 141.45 |
| 10 | 65/F | positive | 9.5/426 | 15 | N | 184 |
| *Patient 1-9 on hydroxyurea, 10 on ruxolitinib | | | |  |  |  |
| ** denotes percentage of allele-burden of JAK2V617F | | | | |  |  |
